# Onychoscopic evaluation of distal and lateral subungual onychomycosis: A cross-sectional study in Lebanon

**DOI:** 10.18502/cmm.5.2.1161

**Published:** 2019-06

**Authors:** Ismael Maatouk, Roger Haber, Nazim Benmehidi

**Affiliations:** 1 Department of Dermatology, Clemenceau Medical Center Affiliated with Johns Hopkins, Beirut-Lebanon; 2 Faculty of Health and Life Sciences, De Montfort University, Leicester LE1-9BH, UK; 3 Saint George Hospital University Medical Center, Faculty of Medicine, University of Balamand, Beirut, Lebanon; 4 Clinic of Dermatology, Ouled Fayet, Algiers, Algeria

**Keywords:** Dermatoscopy, Fungus, Lebanon, Nail, Onychomycosis, Onychoscopy

## Abstract

**Background and Purpose:**

The aim of this study was to evaluate the onychoscopic patterns associated with distal lateral subungual onychomycosis (DLSO) in Lebanon.

**Materials and Methods:**

The present study was conducted on 45 patients with clinical DLSO attending two dermatology clinics in Beirut, Lebanon, between January 2018 and April 2018. The patients were subjected to dermoscopy to identify the onychoscopic patterns.

**Results:**

The DLSO was predominantly associated with white, yellow, and brown color changes (*P<0.05*). Dermoscopic patterns of longitudinal striae (n=31; 68.75%), spiked pattern (n=25; 55.5%), and jagged pattern (n=25; 55.5%) were significantly correlated with DLSO (*P<0.001*). Our findings are in accordance with five previous reports in which dermoscopic findings are discussed in onychomycosis.

**Conclusion:**

It is recommended to perform further studies on homogeneous groups with different clinical subtypes of onychomycosis including patients with suspected traumatic onycholysis or other nail diseases. Identification of onychoscopic patterns would offer the clinicians a quick, simple, and complementary tool for the diagnosis of onychomycosis.

## Introduction

Onychomycosis affects nearly 5% of the population worldwide [1]. It accounts for 40-50% of onychopathies and about 30% of cutaneous fungal infections [2, 3]. In a study conducted on 772 patients in Lebanon in 2006, a positive culture, predominantly with dermatophyte growth, was reported in 54.3% of cases [4]. Onychomycosis is usually diagnosed clinically and can be confirmed by 10-30% KOH examination, fungal culture, and/or nail plate biopsy with periodic acid Schiff (PAS) staining [5, 6]. However, these tests may show false negative results in at least 35% of cases [7].

Onychoscopy is the dermatoscopic evaluation of the nail and its associated structures. This modality facilitates the identification of various patterns and acts as a link between the naked eye examination and nail histopathology, thereby helping physicians and especially dermatologists to reduce unnecessary laboratory examinations. In contrast to other methods, namely fungal culture, direct nail examination, and nail plate biopsy, onychoscopy is a non-invasive, quick, and inexpensive tool for the assessment of onychomycosis. 

Therefore, this technique can be used for the enhancement of the diagnostic accuracy of distal lateral subungual onychomycosis (DLSO). Moreover, in selected cases, it could be a practical and effective diagnostic tool when mycology is not readily available. The aim of this study was to evaluate the onychoscopic patterns associated with DLSO as a morphological type of onychomycosis. Identification of these patterns would offer clinicians a quick, simple, and complementary tool for the diagnosis of onychomycosis.

## Materials and Methods

The present study was conducted on patients with clinical DLSO attending two dermatology clinics in Beirut, Lebanon, between January 2018 and April 2018. Out of 74 clinically suspected cases, the diagnosis of DLSO was confirmed in 45 patients using 20% KOH, fungal culture, or nail biopsy. The patients who were on topical and/or systemic antifungal therapy for the past 3 months were excluded from the study. All patients underwent dermoscopy with a Delta 20T contact dermoscope (Heine’s delta 20 T, Herrsching, Germany) in order to identify the most frequent patterns. 

The observed patterns were defined as jagged (having a non-linear proximal edge of the onycholytic area), spiked (having longitudinal indentations or spikes directed to the proximal fold of the onycholytic area), and striae (having matte pigmentation distributed in striae within the nail plate). After data entry, they were analyzed in SPSS software (version 23). Chi-square test was used to test the association between DLSO type and dermatoscopic pattern. A p-value less than 0.05 was considered statistically significant.

## Results and discussion

The study included 45 patients clinically presenting with DLSO and testing positive for fungal elements by 20% KOH mount, fungal culture, or nail biopsy with PAS stain. The participants were 25 (55.5%) males and 20 (44.4%) females, with a mean age of 43.33 years (range: 24-76 years). The color changes noted in the nail plate included brown (15-33.3%), yellow (14-31.1%), white (10-22.2%), and orange (6-13.3%). However, the black color was not noted. The DLSO was predominantly associated with white, yellow, and brown color changes (*P<0.05*). 

**Figure 1:**
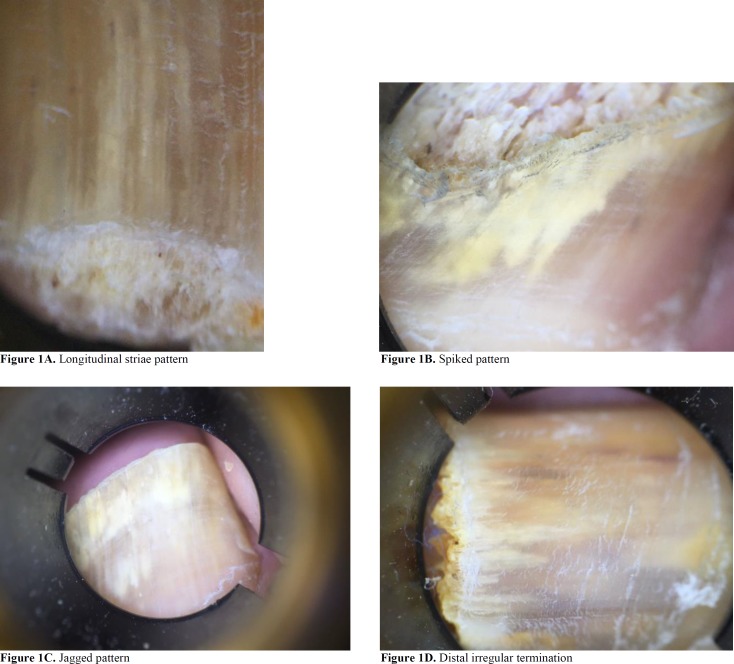
Longitudinal striae pattern as the most common dermoscopic finding

Other changes included brown dots (8-17.7%), pits (4-8.8%), and splinter hemorrhages (2-4.4%). However, there was no statistically significant correlation between these changes and the clinical variants. As shown in Figure 1, the most common pattern noted was longitudinal striae (31-68.75%), followed by spiked pattern (25-55.5%) and jagged pattern (25-55.5%), distal irregular termination (5-11.1%), and linear edge (2-4.4%). Based on the results, the dermoscopic patterns of longitudinal striae (n=31; 68.75%), spiked pattern (n=25; 55.5%), and jagged pattern (n=25; 55.5%) statistically correlated with DLSO (*P<0.001*).

**Table 1:** Summary of onychoscopic patterns found in distal lateral subungual onychomycosis

**Location**	**Onychoscopic finding**	**Pathophysiological explanation**
Proximal edge of the onycholytic area	Jagged; never linear	Proximal progression of dermatophytes along the horny layer of the nail bed
Proximal edge of the onycholytic area	Longitudinal indentations directed to the proximal fold as “spikes”	
Nail plate	Irregular matte pigmentation distributed in “striae”	Progression of dermatophytes along the nail plate, showing changes in coloration secondary to colony formation, flakes, or subungual debris.

**Table 2:** Comparison of onychoscopic patterns in distal lateral subungual onychomycosis obtained in this study with reports

	**Our study (n=45 DLSO)**	**Piraccini et al., 2013**	**De Crignis et al., 2014**	**Jesus Silva et al., 2015**	**Yadav et al., 2016**	**Chetana et al., 2018**	**Correlation with clinical type**
**Longitudinal striae**	31 (68.75%)	32 (86.4%)	267 (79.46%)	42 (44.68%)		86 (62.2%)	DLSO (*P<0.001*)
**Spiked pattern**	25 (55.5%)	37 (100%)		17 (43.59%)	21 (58.3%)	71 (52.5%)	DLSO (*P<0.001*)
**Jagged pattern**	25 (55.5%)	37 (100%)				53 (39.2%)	DLSO (*P<0.001*)
**Linear edge (traumatism)**	2 (4.4%)	13 (100%) – Cases of traumatic onycholysis		13 (38.24%)		5 (3.7%)	
**Distal irregular termination**	5 (11.1%)			26 (38.81%)		29 (21.5%)	

In our study, longitudinal stria was the predominantly observed pattern (n=31, 68.75%) in DLSO, which is in accordance with the previous reports [8-12]. This pattern may be the result of the dermatophyte invasion along the nail plate; in addition, discoloration may be secondary to colony formation, flakes, or subungual debris [9, 10]. The second predominant patterns in DLSO were spiked and jagged patterns, each of which occurred in 25 (55.5%) samples.

Spiked pattern was described by Yadav et al. as white, irregular streaks demarcating the area of onychomycosis in cases with DLSO [11]. Piracinni et al. stated that these spikes are exclusively observed in DLSO cases [8]. The jagged pattern corresponds to the proximal progression of dermatophytes along the horny layer of the nail bed in the form of longitudinal ridges. The remaining patterns did not have any correlation with the different clinical types in our study. These findings are summarized in Table 1. In addition, Table 2 presents a comparison between our results and the previous reports.

The linear edge pattern without any indentations has been consistently associated with traumatic onycholysis [8, 9, 12]. This dermatoscopic finding is used to differentiate onychomycosis from traumatic onycholysis [8, 9, 12]. In our study, the presence of this pattern in DLSO (n=2, 4.4%) was not statistically significant and may be attributed to traumatic onycholysis preceding onychomycosis.

This is the first study conducted in the Lebanese population and the Middle East demonstrating dermoscopy as an important adjunctive tool in the evaluation of nail diseases in general and onychomycosis in particular. However, this method presents many limitations since it fails to determine the type of causative fungus. Onychoscopy should be regarded as a quick, simple, and complementary tool for the diagnosis of onychomycosis. 

However, this technique should not replace the standard tests when needed. Furthermore, our study population did not include homogeneous groups with different clinical subtypes of onychomycosis and consisted of onychoscopic patterns extensively limited to only DLSO. Additionally, our study included cases which were positive based on one of the methods of direct examination, culture, or nail biopsy. 

## Conclusion

To the best of our knowledge, this is an additional study to the five previously published reports in which dermoscopic patterns in onychomycosis have been discussed. Our findings regarding the frequent longitudinal striae, jagged, and spiked patterns observed in DLSO are in accordance with the previous descriptions. It is recommended to perform further studies using homogeneous groups with different clinical subtypes of onychomycosis and including patients with suspected traumatic onycholysis or other nail diseases. Identification of these patterns would offer clinicians a quick, simple, and complementary tool for the diagnosis of onychomycosis.
